# Comparison of shear bond strength of CAD/CAM and conventional heat-polymerized acrylic resin denture bases to auto-polymerized and heat-polymerized acrylic resins after aging

**DOI:** 10.4317/jced.59097

**Published:** 2022-01-01

**Authors:** Masoumeh Taghva, Shabnam Enteghad, Ali Jamali, Mina Mohaghegh

**Affiliations:** 1 Assistant Professor, Department of Prosthodontics, School of Dentistry, Shiraz University of Medical Sciences, Shiraz, Iran; 2 Postgraduate Student, Student Research Committee, Shiraz University of Medical Sciences, Shiraz, Iran; 3 Undergraduate Student, Student Research Committee, Shiraz University of Medical Sciences, Shiraz, Iran

## Abstract

**Background:**

The aim of this study was to investigate the shear bond strength of CAD/CAM and conventional heat polymerized acrylic resin denture bases bonded to self-cured and heat-cured acrylic resins after aging.

**Material and Methods:**

A total of 40 cubic specimens were fabricated from conventional heat-polymerized and CAD/CAM denture base resins. Denture base resin specimens in each group were divided into two subgroups (n=10) in which they were bonded to either a heat-cured (HC) or a self-cured (SC) reline resin. Subsequently, the specimens were subjected to thermocycling. Then the shear bond strength (SBS) of specimens was measured using the universal testing machine. After testing, modes of failure were examined using light microscopy. The results were submitted to statistical analysis.

**Results:**

Mann-Whitney test showed that in each group of denture base materials, specimens bonded to HC reline resin had significantly higher SBS than those bonded to SC reline resin (*P*<0.001). Conventional denture base bonded to HC resin exhibited the highest value of SBS. There was no statistically significant differences between the SBS of HC reline resin bonded to conventional and CAD/CAM with regards to SBS (*P*=0.218). However, the SBS of SC reline resin was significantly higher when bonded to CAD/CAM compared to conventional denture base resin (*P*<0.001).

**Conclusions:**

Heat-cured reline resin showed higher shear bond strength to both CAD/CAM and conventional heat-polymerized denture resin in comparison to self-cured reline resin. Although there was no difference between the bond strength of heat-cured reline resin to CAD/CAM and conventional denture base, self-cured reline material produced stronger bond with CAD/CAM denture base.

** Key words:**CAD/CAM, shear bond strength, reline, denture base resin.

## Introduction

Acrylic resin dentures often need to be relined due to progressive residual ridge resorption which is associated with poor adaptation of the denture to the oral tissue, lack of stability, and patient’s dissatisfaction (1,2). In addition, acrylic resin dentures are susceptible to fracture and might need to be repaired (3). Denture relining and repairing materials could be used to enhance the prosthesis retention and support and bring function and esthetics back to patient. Several materials have been used to reline or repair removable dentures, including auto-polymerized, heat-polymerized, visible light-polymerized and microwave-polymerized acrylic resins (4,5). Among these, the use of chairside auto-polymerizing resins and heat cure resins which need laboratory process is the most popular. Chairside auto-polymerizing resins allow for a simple and quick repair. Also, they show good resistance and acceptance by patients. On the other hand, heat-polymerizing resins display higher durability, proper flow, lower porosity and greater detail reproduction (6,7).

To ensure a satisfactory outcome, a reliable bond between the denture base material and reline resin is important. A weak bond is associated with reduced mechanical properties of the denture, bacteria accumulation, staining or complete delamination of both the denture base resin and reline material (5,8). Clinically, the stresses applied to the interface of the two materials, leading to bond failure, are most closely related to shear and tear (9). Therefore, shear bond strength test, which exerts the load directly to the interface of denture base and liner, has been extensively used to investigate bond strength of reline materials (4,10-12). It has been shown that bond strength depends on the chemical compositions of both the reline/repair material and the denture base polymer (10,13). Other factors affecting the bond strength are surface treatment and thermal cycling (4,14,15). Thermocycling in an aqueous environment has been widely used to replicate temperature changes in the wet oral environment and to simulate the effects of aging on the properties of materials including bond strength (16).

Since the fabrication of the first removable dentures using poly methyl methacrylate (PMMA), there were no major changes in fabrication techniques until computer-aided design and computer-aided manufacturing (CAD/CAM) techniques was introduced in the 1990s (17). The CAD/CAM manufacturing process of the complete denture begins with the use of optical scanning technology to record the clinical information from the patient and using digital softwares to design the denture (CAD). The next step is the automated manufacturing process (CAM) that can be subtractive (computer controlled milling) or additive (rapid prototyping), the former being more commonly used (18). Advantages of CAD/CAM methods to fabricate dentures are lower clinical chairtime and fewer number of visits, digital archiving and more desirable clinical and patient-centered outcomes (19,20). CAD/CAM denture bases are milled from prefabricated acrylic resin blocks that were previously polymerized under high temperature and pressure, resulting in superior mechanical and physical properties of the denture base including less monomer release, fewer microporosities, higher retention and increased toughness, flexural strength and surface hardness (18,21).

Like other dentures, after a period of usage, the CAD/CAM dentures may need relining due to continuous residual ridge resorption or they may need to be repaired due to the misuse of denture by patient. However, since CAD/CAM is an almost new technique for fabrication of dentures, there is little knowledge about the bonding properties of CAD/CAM acrylic denture base materials to acrylic resins. One study evaluated the tensile bond strength of three resilient reline materials bonded to denture base resins, and concluded that resilient reline materials showed the lowest tensile bond strengths when bonded to CAD/CAM denture bases in comparison with auto-cured and heat-cured denture bases (16). A recent study showed that CAD/CAM subtractive denture bases exhibit lower flexural and tensile bond strength to hard reline materials in comparison with the conventional denture bases (22). The aim of the present study was to investigate the shear bond strength of auto-polymerized and heat polymerized acrylic resins to CAD/CAM and conventional heat-polymerized acrylic resin denture bases after aging. The null hypothesis was that shear bond strength of self-cured and heat-cured acrylic repair resin to CAD/CAM denture base is not different from shear bond strength of these repair resins to conventional heat-cured denture base.

## Material and Methods

This research has been approved by the ethics committee of Shiraz University of Medical Sciences (IR.SUMS.DENTAL.REC.1399.055).The materials used in the present study were all denture base resins with Polymethyl Methacrylate (PMMA) base. The products names, material types, powder/liquid proportions and manufacturers are summarized in Table 1.

**Table 1:**
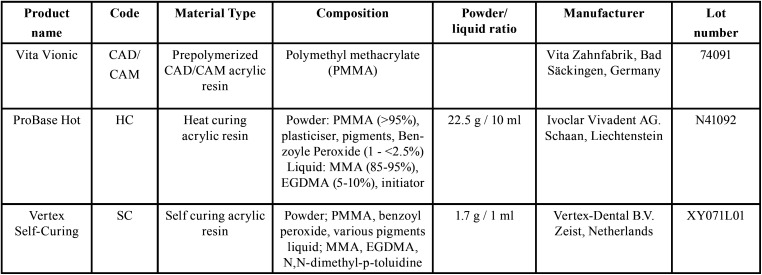
Properties of the materials used in the study.

-Specimen preparation

**Figure 1:**
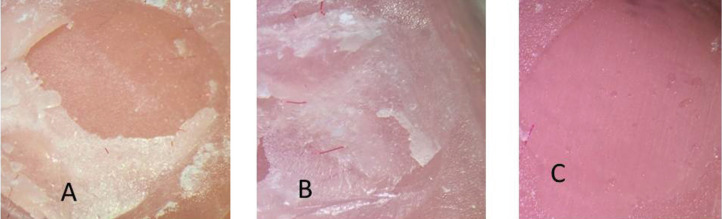
Shear bond strength (SBS) testing of the specimens using Universal testing machine.

For the preparation of heat-cured acrylic resin (HC) specimens, 20 plastic dies with dimensions of 10 * 10 * 20 mm3 were fabricated by a 3D printer (Quantum 2025 desktop 3D printer, Persia 3D printer Co., Tehran, Iran) (Fig. 1). According to the manufacturer’s instructions, plaster with the powder to liquid ratio of 3.5:1 was prepared and poured in the first half of flask. Then, the die was put half in the plaster. After the initial hardening of plaster, a layer of biofilm was applied to the surface of the plaster and the die, and then the flask was filled with plaster. Then, the flask was opened and the die was taken out of the plaster. According to the manufacturer’s instructions, HC resin was prepared and packed into the mold created in the flask at the doughy stage. Then, the flask was processed in a water bath at 70°C for 9 hours. The flask was subsequently bench cooled and opened. After removing the flashes and trimming the specimens, 20 specimens with the same size as the original die were obtained.

In order to prepare 20 CAD/CAM acrylic resin specimens, an acrylic block of CAD/CAM with a thickness of 30 mm and a diameter of 98 mm was prepared. The cubic shaped specimens with dimensions of 10 * 10 * 20 mm3 were designed by CAD and the block was cut by a milling machine (ARUM 5X-400, Doowon ID CO., Ltd., Daejeon, Korea).

The bonding surface of all HC and CAD/CAM specimens was sandblasted with 110 micron Aluminum oxide particles at 2 bar for 10 seconds. The specimens were immersed in tap water for 72 hours. Afterwards, they were dried and their surface was cleaned with ethyl alcohol to ensure a clean bonding interface.

Denture base resin specimens in each group were divided into two subgroups (n=10) based on the acrylic resin material to which the denture base specimen was bonded. According to that, the HC denture base resin was bonded to HC resin in group A1 and to self cure acrylic resin (SC) in group A2. The CAD/CAM denture base resin was bonded to HC resin in group B1 and to SC resin in group B2.

To bond the specimens with HC resin (groups A1 and B1), cylindrical plastic dies with dimensions of 10 mm and a diameter of 6 mm were fabricated by a 3D printer (Quantum 2025, Iran). The plastic dies were buried inside silicon putty (Coltene Speedex Putty; Coltene AG, Altstatten, Switzerland) and after putty setting, the dies were removed. The empty place of the plastic die was filled with onlay wax and after the wax cooled, they were taken out of the putty. Following that, the wax cylinders were placed on the surface of 10 HC samples and 10 CAD/CAM samples. The samples were placed in a flask. Plaster was poured to the height of the wax cylinders. After the plaster hardened, a layer of biofilm was applied to the plaster surface. The flask was then filled with plaster and after the setting of the plaster, the wax was burned out and the flask was opened. HC resin was prepared according to the manufacturer’s instruction and was placed in the empty space inside the plaster and then the flask was pressed at 100 pounds for 30 minutes. Subsequently, the flask was processed in a water bath at 70°C for 9 hours. Then, it was slowly cooled and opened. The plaster around the specimens was gently removed with a laboratory handpiece. The specimens were placed in a thermocycling device (Vafaee Company, Iran) for 1500 cycles between 5˚C and 55˚C.

To bond specimens with SC resin (groups A2 and B2), similar cylindrical plastic dies were fabricated and placed on the prepared surface of 10 CAD/CAM specimens and 10 heat-cured specimens. Following that, the specimens and the die on top them were embedded in silicon putty. After silicon putty setting, dies were removed from the putty and a mold was created. The SC resin was prepared according to the manufacturer’s and placed at the dough stage inside the space created in the putty. Afterwards, a glass slab was placed on the samples and the weight of 1 kg was placed on the slab for 30 minutes. Twenty-four hours after the setting of SC resin, the putty was removed and samples were placed in the thermocycling device for 1500 cycles between 5˚C to 55˚C.

-Shear bond strength test

Shear bond strength (SBS) test for 40 specimens was performed through a Universal testing machine (Zwick_Roell Zo20, Zwick, U1m, Germany) at a crosshead speed of 1 mm/min until fracture occurred. To determine the bond strength in MPa, the load was divided to the cross sectional area of adhesion. Specimen were observed with a stereomicroscope (Bestscope BS-3060; Best Scope, China) at 25× magniﬁcation to assess the mode of failure. Upon the sight of the residual of repair resin in less than 30% of the bonding area, it was counted as adhesive and the residual of repair resin appearing in 30% to 70% of bonding area was counted as mixed. Besides, if the residual of repair resin was seen in more than 70% of bonding area, it was counted as cohesive failure.

**Figure 2:**
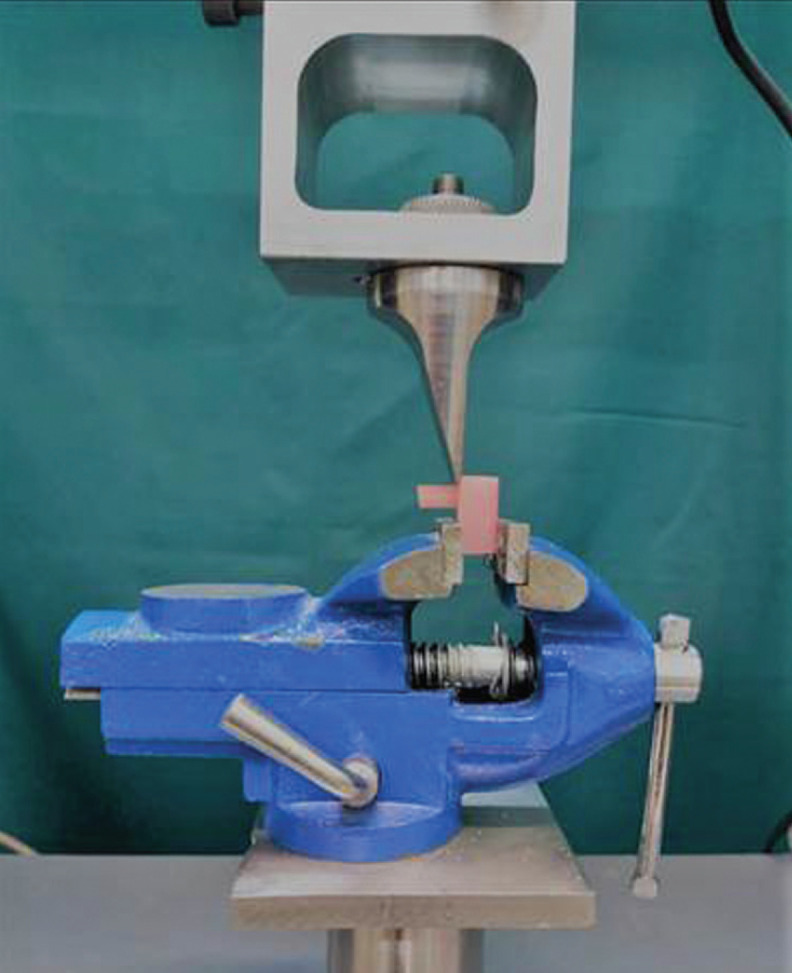
Mean shear bond strength values (MPa) for all groups.

Data were analyzed using IBM SPSS version 22 (SPSS Inc., IL, USA). Normality of data was assessed by Kolmogorov-Smirnov and data were analyzed by Kruskal-Wallis and Mann-Whitney U test at the significance level of 0.05, (Fig. 2).

## Results

The mean and standard deviation (SD) of the SBS values and *p-value*s are listed in Table 2. Kruskal-wallis test showed significant difference between the SBS of the two reline materials bonded to HC and CAD/CAM denture bases. The results of Mann-Whitney U test for sub-group analysis revealed that A1 (HC-HC) and B1 (CAD/CAM-HC) had significantly higher SBS values than A2 (HC-SC) and B2 (CAD/CAM-SC), respectively (*p-value*<0.001). In addition, SBS values for B2 was significantly larger than A2 (*p-value*<0.001). There was no statistically significant difference between A1 and B1 with regards to SBS (*p-value*=0.218).

**Table 2:**
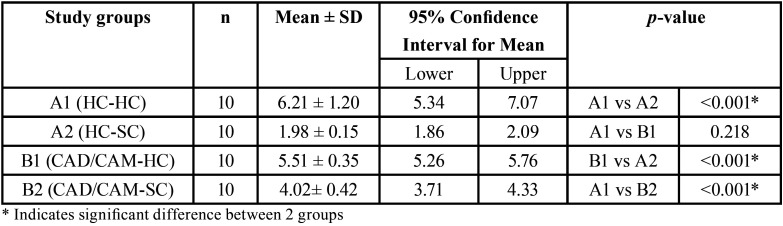
Mean and standard deviation (SD) for shear bond strength values (MPa).

**Figure 3:**
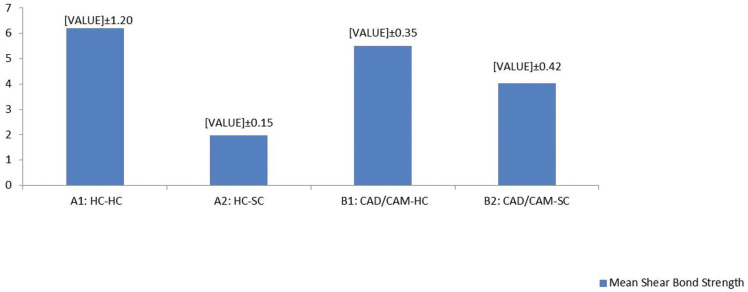
Different modes of failure observed in the study; A. Cohesive failure B. Mixed failure C. Adhesive failure. Images A, B, and C belong to specimens from the groups HC-HC, CAD/CAM-HC, and HC-SC, respectively.

Regarding the modes of failure, cohesive followed by mixed failure were the most frequent failure modes observed in all study groups, except for group A2 (HC-SC) in which adhesive failure accounted for the highest percentage of failure modes. Table 3 demonstrates the failure modes observed in the fracture site and their percentages among each study group. Stereomicroscopy images of fractured surfaces are represented in Figure 3.

**Table 3:**
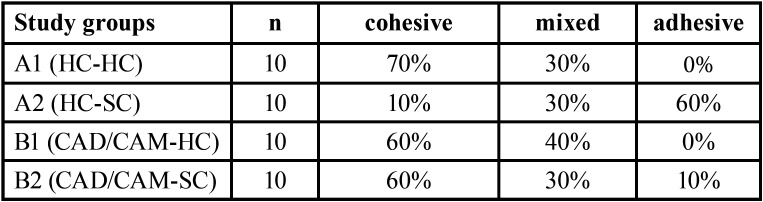
Variable Mode of failures and their percentages.

## Discussion

The null hypothesis was rejected as the result demonstrated that the SBS was significantly different among the study groups. The highest SBS was observed in group A1 (HC-HC). This could be due to the fact that in these specimens the denture base and the reline resin were the same material. It has been well reported that when the reline material has a chemical composition identical or similar to the original denture base, the highest values of SBS, flexural strength and fracture resistance are achieved (23-26).

Results showed that HC resin liners produced a significantly higher SBS when bonded to both types of denture bases (HC and CAD/CAM) compared with the SC reline material. Successful bonding between the denture reline and base polymers depends on the effective penetration of polymerizable monomers from the reline material into the denture base network (10,25,27). The higher SBS of HC reline resins in comparison to SC resin may be due to the fact that the heat-cured acrylic resin is processed under a higher temperature and has a longer polymerization time which leads to higher diffusion rates of the monomers into the denture base resin (28), and to higher degree of conversion which leaves less residual monomer content (29). Unpolymerized monomer in the self-cured resin has a plasticizing action within the polymer matrix which leads to porosity formation that will reduce the mechanical properties including the bond strength of reline resin to denture base (30).

Better mechanical properties of heat-polymerized reline resins, including bond strength, compared with autopolymerizing resins, have been confirmed by other investigations. AlQahtani *et al*. concluded that the PMMA denture bases repaired with heat-polymerizing resins resulted in a significantly greater flexural strength compared to autopolymerizing and light-polymerizing resins (26). Leong *et al*. (31) stated that the transverse strength of denture bases relined with heat-cured resins was up to 75% of the original material, while acrylic resin denture base relined with self-polymerizing resin materials exhibited approximately 60% of transverse strengths of the original denture base. In the contrary, Agarwal *et al*. (32) and Rached *et al*. (33) reported a similar transverse strength for heat-polymerized resin and autopolymerized resins.

The mean SBS for HC denture base resin bonded to SC resin was the lowest among the four study groups (1.98 MPa). However, in previous studies (4,12,28), higher values of SBS were reported. The difference could be due to the subjecting of the specimens to thermocycling procedures in our study, which was not performed in the mentioned studies.

The results of the present study indicated that SC resin had significantly higher SBS when bonded to CAD/CAM denture base compared to HC denture base. In the case of SC reline material bonded to HC denture base, the bond strength could have declined due to the water absorption during thermocycling. The water molecules absorbed in the resin polymer network can act as plasticizers, and enhance its softness and the material elasticity consequently decreasing the bond strength (16,28). As reported previously, CAD/CAM PMMA-based denture resins have lower porosities and voids and stronger cross linkage between polymer chains, leading to reduced water absorption (34,35), which may explain the higher bond strength of CAD/CAM samples to SC resin. Another factor that may be related to this finding is the possible differences in thermal expansion coefﬁcient (CTE) between the denture base resin and the reline material. Differences in CTE of materials lead to different degrees of shrinkage and expansion, which can exert a cyclic stress at the interface and increase the fatigue of bond during thermocycling (27,36). Furthermore, the preparation method could have affected CAD/CAM denture resin and conventional heat-cured acrylic resin differently. More investigations are required to evaluate the CTE of these materials and the effect of surface treatment methods on the bond strength.

This result is in contrast with the findings of the study by Wemken *et al*. which reported higher flexural and tensile bond strength of conventional denture base to hard chairside liners in comparison to CAD/CAD denture base after thermocycling. Authors explained this finding by the higher residual monomer contained in the conventional denture base compared to CAD/CAM denture base, which resulted in higher bond with the hard chairside reline material (22). Regarding the adhesion of CAD/CAM and conventional denture bases to other materials, Choi *et al*. compared the bond of denture characterizing composites to heat-polymerized and CAD/CAM denture base resins and demonstrated better bond strengths of characterizing composites with CAD/CAM bases under thermocycling (37). The same authors evaluated the bond strength of acrylic denture teeth (27) and resilient denture liners (16); they reported improved bond strengths of these materials to heat-polymerized compared with CAD/CAM denture base. It must be considered that making comparison of these studies is difficult due to their different experimental design, combinations of materials, regimens of thermocycling, and different bond strength tests.

Bond failure of denture base and reline material can be adhesive, cohesive or mixed. Adhesive failure, which is considered the least accepTable, indicates that the interface of the reline material and the denture base resin lacks strength, whereas in cohesive failure, the bond strength is greater than the resistance of each material alone and is adequate (8). This is in accordance with the modes of failure observed in the present study. With higher SBS values, higher percentages of cohesive and mixed failures were also evident, whereas in HC-SC specimens with the lowest SBS, mostly adhesive failure was observed.

One of the limitations to the present study was the fact that the samples were analyzed only after thermocycling. The effects of thermocycling and various surface treatments on the bond strength of acrylic resins to conventional and CAD/CAM denture bases should be investigated in future studies. Furthermore, it is imperative to investigate the physical characteristics of reline and repair materials in clinical use and the effect of the oral environment on such properties. As it was highly unlikely to completely replicate oral environment due to the *in vitro* nature of the experiment, the final assessment must be carried out in clinical situations.

## Conclusions

Within the limitations of this study, it was concluded that heat polymerizing acrylic denture liners have greater shear bond strength, compared to autopolymerizing acrylic denture liners, when bonded to both conventional and CAD/CAM denture bases, with no significant difference between the two denture base resins. However, there is a significant difference between autopolymerizing acrylic resin bond strength to CAD/CAM and conventional denture base. Autoplymerizng reline material seems to produce stronger bond with CAD/CAM denture base.
